# Susceptibility of livestock to SARS-CoV-2 infection

**DOI:** 10.1080/22221751.2021.2003724

**Published:** 2021-11-22

**Authors:** Angela M. Bosco-Lauth, Audrey Walker, Lauren Guilbert, Stephanie Porter, Airn Hartwig, Emma McVicker, Helle Bielefeldt-Ohmann, Richard A. Bowen

**Affiliations:** a Department of Biomedical Sciences, Colorado State University, Fort Collins, CO, USA; b School of Chemistry & Molecular Biosciences and Australian Infectious Diseases Research Centre, The University of Queensland, St Lucia, Australia

**Keywords:** SARS-CoV-2, livestock, cattle, sheep, goat, alpaca, rabbit, horse

## Abstract

We report pilot studies to evaluate the susceptibility of common domestic livestock (cattle, sheep, goat, alpaca, rabbit, and horse) to intranasal infection with SARS-CoV-2. None of the infected animals shed infectious virus via nasal, oral, or faecal routes, although viral RNA was detected in several animals. Further, neutralizing antibody titres were low or non-existent one month following infection. These results suggest that domestic livestock are unlikely to contribute to SARS-CoV-2 epidemiology.

The SARS-CoV-2 virus, causative agent of COVID-19 disease, is a known zoonosis, with natural infections occurring in non-human primates, felids, canids, and mink, and experimental infections proving susceptibility in a variety of other species including wildlife and laboratory animals [1–6]. The virus is thought to have originated from wildlife, and possibly introduced to humans in a live animal market in Wuhan, China in December 2019, but to date there is no consensus on the species of origin. While bats are a likely source of this emerging virus given the sequence similarity to other bat coronaviruses, experimental studies in bats thus far do not confirm this theory [7–9]. Thus, the reservoir(s) of SARS-CoV-2 remain to be identified, and scientists must resort to serosurveillance and experimental infection studies to elucidate possible reservoir hosts.

Determining the host range, pathogenesis, and transmissibility of an emerging pathogen is immensely important in order to better understand the epidemiology of the resulting disease, and target surveillance and mitigation efforts. Furthermore, it is important to determine both the risk of zoonotic disease transmission (infection of humans by animals) and reverse zoonosis (infection of animals by humans) of those species that are in close contact with humans. Early in the pandemic, cases of dog and cat infections were reported, primarily animals with SARS-CoV-2 infected owners, and these reports quickly instigated efforts to determine how pets and other domestic animals would respond to SARS-CoV-2 infection, and what risk these animals might play in leading to more human exposure to the virus [1]. Here, we report experimental studies to assess baseline susceptibility to infection by SARS-CoV-2 in several common livestock animals.

The approach described herein was to intranasally inoculate animals from representative livestock species (cattle, sheep, goats, alpaca, rabbits, and one horse), monitor for clinical disease, sample for viral shedding (nasal/oral, rectal), measure viral titres in respiratory organs from acute-stage necropsies, and determine antibody production over the course of one month in most species (Table 1). Baseline serum samples were obtained and screened for existing antibodies using plaque reduction neutralization tests (PRNTs) as previously described [6]; all animals were seronegative at the onset of the study. Intranasal inoculation was performed via dropwise instillation of between 4.5 and 7 log_10_ plaque-forming units (pfu) SARS-CoV-2 virus strain 2019-nCoV/USA-WA1/2020, obtained from BEI Resources (Manassas, VA, USA) and passaged three times in Vero cells. Thermal microchips were used to evaluate body temperature for the duration of the studies, and nasal and rectal swabs were collected on days 1–7 for all animals except rabbits, from which oral and rectal swabs were collected, and alpacas, from which only nasal swabs were obtained. One or two animals from the following groups were sacrificed and necropsied on day 3 post-infection (DPI): cattle, sheep, goat, rabbit, and horse, and the remainder were euthanized 28 days post-infection. Virus isolation from swabs and tissues was attempted using plaque assays on Vero cells as previously described and real-time RT–PCR was performed on 3 DPI samples and tissues [6,10]. Tissues (turbinates, soft palate, mandibular lymph node, trachea, lung, heart, liver, spleen, kidney, small intestine) collected at 3 DPI were also fixed in formalin for histopathological evaluation by a veterinary pathologist. Terminal sera were tested for virus-neutralizing antibody by PRNT.

**Table 1.** Species tested and summary of virus isolation, RT-PCR and PRNT results

Species	# animals	Dose	Samples collected for VI	Virus isolation	RT-PCR (+/total)	14 DPI PRNT90 (range)
Cattle	3	5.4	Nasal, Rectal, Tissues	1/3*	1/3	<10
Goat	3	5.4	Nasal, Rectal, Tissues	0/3	2/3	<10-10
Sheep	4	4.5	Nasal, Rectal, Tissues	0/4	0/4	<10-10
Rabbit	4	4.7	Oral, Rectal, Tissues	0/4	1/4	<10
Horse	1	6.3	Nasal, Tissues	0/1	0/1	NT
Alpaca	2	7	Nasal	0/2	0/2	<10

* Plaques from tracheal tissues confirmed as SARS-CoV-2 via RT-PCR

NT = Not tested

In this study, none of the animals shed detectable infectious virus during the course of the study, while several individual animals (1 calf, 2 goats, and one rabbit) had RT–PCR positive nasal and/or oral swabs, which suggests that these animals may be minimally permissive to infection. Live virus was isolated from the trachea of one calf necropsied on 3 DPI, but no other tissues were positive in that animal, suggesting local infection of the upper respiratory tract during acute infection. The single horse used in this experiment did not shed virus nor was infectious virus detected in any organs at the time of necropsy; serology was not performed on this animal. Interestingly, while several animals developed low-level neutralizing antibodies within 14 days of infection (Table 1), the majority were seronegative by 28 DPI. None of the animals necropsied on 3 DPI had histopathological lesions consistent with SARS-CoV-2 infection, including the single calf with the infected trachea. Furthermore, none of the animals displayed any clinical signs of disease or fever following inoculation. These results are consistent with other livestock studies demonstrating low level viral replication in pigs, cattle and rabbits [11–13]. To the author’s knowledge, this is the first report of SARS-CoV-2 experimental infection in goats, sheep, alpaca, or horses. Importantly, the lack of shedding in alpacas, which are highly susceptible to Middle Eastern Respiratory Syndrome Coronavirus (MERS-CoV), suggests camelids are unlikely to serve as a source for a recombination event between MERS-CoV and SARS-CoV-2 [14]. Overall, these results suggest that domestic livestock species are poorly competent hosts for SARS-CoV-2 and are unlikely to contribute to disease transmission or epidemiology.

An obvious limitation of this study is the very small sample size; however, considering that other highly susceptible animals [2–4, 6] are readily infected, we believe these results provide sufficient evidence to exclude the species evaluated herein from the highly susceptible category. While all species described herein were inoculated with an early isolate of SARS-CoV-2, we don’t have any reason to believe that newer human-adapted variants are any more likely to replicate in these poorly susceptible species. Rather, we posit that domestic livestock are low-risk for participating in a spill-over event or reverse zoonosis, as predicted by studies comparing host range using ACE2 protein sequence, in which binding likelihood was predicted based on sequence homology to human ACE2 [15], and the lack of reports of any such species becoming naturally infected in the first 18 months of the pandemic lends credibility to this position. While there is much yet to be learned about the role of animals (wild or domestic) in the COVID-19 pandemic, including how the emergence of novel variants might impact non-human species, with each new study investigating the potential for animals to serve as reservoirs, we learn more about how SARS-CoV-2 behaves, and, perhaps, get closer to uncovering the answer to its origin.
